# Interval compressed vincristine, doxorubicin, cyclophosphamide alternating with ifosfamide, etoposide in patients with advanced Ewing’s and other Small Round Cell Sarcomas

**DOI:** 10.1186/2045-3329-2-12

**Published:** 2012-09-21

**Authors:** Jeremy Whelan, Atia Khan, Anand Sharma, Christian Rothermundt, Palma Dileo, Maria Michelagnoli, Beatrice Seddon, Sandra Strausss

**Affiliations:** 1 Department of Oncology, The London Sarcoma Service, University College Hospital London NHS Foundation Trust, 1st Floor Central, 250 Euston Road, London NW1 2PG, UK

**Keywords:** Ewing’s sarcoma, Desmoplastic small round cell tumour, Chemotherapy, Sarcoma

## Abstract

**Background:**

To evaluate tolerability and maintenance of dose intensity of 2 weekly treatment with vincristine, doxorubicin, cyclophosphamide alternating with ifosfamide, etoposide (VDC/IE) in patients with advanced small round cell sarcomas including Ewing family tumours (EFT), desmoplastic small round cell tumours (DSRCT) and undifferentiated high grade round cell sarcomas (UHGRCS).

**Methods:**

Retrospective review of 16 patients treated at a single centre with VDC/IE. Dose received, treatment delay, toxicity and clinical outcome were recorded for each cycle up to a maximum of 14 cycles.

**Results:**

A total 193 cycles of VDC/IE were administered to 10 patients with EFT, 4 with DSRCT and 2 with UHGRCS. Median age was 22 years with 75% over 18 years. Metastases were present in 14 patients. The mean duration of each cycle was 16.7 days. Febrile neutropenia occurred in 14 % of cycles, and grade 3/4 haematologic toxicity including anaemia and thrombocytopenia in 16 % and 11 % of cycles respectively. Seven patients had a dose reduction. Five patients discontinued VDC/IE early due to toxicity.

**Conclusions:**

This schedule of VDC/IE is feasible in patients with EFT and DSRCT including adults and those with metastases. Its comparison with other standard regimens for these diseases is justified.

## Background

The Ewing’s family of tumours (EFT) are the second most common malignant bone tumour seen in children and young people [1,2]. Histologically, they are characterized by small round blue cells with immunohistochemical staining for CD99 and neural markers. A reciprocal translocation between chromosomes 11 and 22 is evident in more than 85% of these tumours [3,4]. The family of small round blue cell sarcomas also includes desmoplastic small round cell tumour (DSRCT), a rare soft tissue sarcoma characteristically presenting in young males with extensive multifocal intraabdominal disease. Similar chemotherapy approaches to those utilized for EFT are used, albeit with less satisfactory results as progression and ultimately death due to disease is almost universal [5-7].

Since the introduction of multimodality treatment in EFT, survival has improved from 10% to 75 % in patients with localized disease [8-11]. Since the 1980’s, chemotherapy regimens have evolved both in Europe and the United States to include anthracyclines and alkylating agents with only modest variations in dose and schedule [9,10,12-15]. To contend with a lack of recent survival improvement or new agents with major activity, investigators have concentrated on investigating the benefits of scheduling and dose intensity.

The current European Ewing tumour Working Initiative of National Groups 1999 (EURO-EWING 99) study has enrolled over 3,200 patients in a study evaluating an intensive induction regimen (VIDE, vincristine, ifosfamide, doxorubicin and etoposide) and, in selected cohorts of randomized patients, high dose chemotherapy with stem cell rescue. Toxicity associated with VIDE chemotherapy is substantial. For example, neutropenia and related fever is reported in 60.8% and 65.8% of courses respectively [16].

In North America, alternative approaches to dose optimization have been explored. The Children’s Oncology Group (COG) study, INT-0154, using a regimen of vincristine, doxorubicin, cyclophosphamide alternating with ifosfamide and etoposide (VDC/IE), randomized patients to an increased treatment intensity by higher doses of cyclophosphamide and ifosfamide and a decreased length of treatment to 11 cycles over 30 weeks in the test arm compared with a standard 17 cycles over 48 weeks but with equivalent total drug doses in each arm. There was no survival improvement but more toxicity in the dose intense arm [17]. In contrast a survival advantage has been reported in the preliminary results from a further COG study of patients with localized EFT, AEWSOO31, randomizing between a standard three weekly schedule and an ‘interval compressed’ two weekly schedule of VDC/IE, the latter made possible by growth factor support [18]. The mean cycle durations were 18.5 and 23.3 days for the two and three weekly cycles respectively. Event free survival at 3 years was significantly extended in the two weekly arm, 76% vs. 65%, p = 0.028. Toxicity was similar in the two treatment arms but with the reported frequency of febrile neutropenia and other major toxicities apparently lower than those seen with VIDE. Interval compressed VDC/IE has consequently been adopted as the standard of care for future studies of EFT by COG.

There are significant advantages to defining a standard chemotherapy regimen for EFT, not least as a platform for testing new agents in an international setting, which is essential in studying rare cancers. Additional goals for all investigators are reducing both short and late toxicity in a young population of whom approximately two thirds will achieve long term survival, and of course limiting the treatment burden in those with poor prognostic factors. Whether interval compressed VDC/IE is less toxic than VIDE is speculative in the absence of comparative data. Furthermore, the AEWS0031 study was limited to patients with localised disease and had only a small proportion of patients aged over 20 years, so it may not be appropriate to extrapolate the data to the entire EFT population.

In preparation for a planned randomized comparison in Europe of first line treatment for EFT between VIDE and VDC/IE, we retrospectively examined the feasibility of interval compressed VDC/IE in patients with metastatic EFT. In recognition that less toxic treatment would be appropriate for older patients [19] and those with a poor prognosis, we did not restrict the age of our cohort, and also included those with DSRCT. This latter group responds temporarily to chemotherapy regimens used in EFT but with a 5-year survival of only 15% [20]. Our institutional standard for DSRCT and UHGRCS includes treatment with VIDE. Identifying a more tolerable but effective regimen is a priority.

## Materials and methods

### Patient selection

A consecutive series of patients were treated with interval compressed VDC/IE if either ineligible for the EURO-EWING 99 study (including those with extrapulmonary metastases), or were newly diagnosed with DSRCT or UHGRCS. A histological diagnosis was required in all patients. Staging included plain X-rays and magnetic resonance imaging (MRI) or computed tomography (CT) scan of the primary site, chest CT scan and whole body technetium (^99m^ Tc) bone scan were performed to confirm the presence of metastases. Routine assessments of renal and cardiac function were conducted at baseline, during and after therapy. Institutional ethics guidance was followed.

### Chemotherapy

The VDC/IE regimen was alternate 14 day cycles of VDC (vincristine 1.4mg/m^2^, maximum 2mg, doxorubicin 75mg/m^2^ and cyclophosphamide 1200mg/m^2^ administered over 2 days) and IE (ifosfamide 9gm/m^2^, etoposide 500mg/m^2^ fractionated over 5 days). Patients had a full blood count measured on the day prior to the cycle due date and treatment was given if the neutrophil count was greater than 1 × 10^9^/l, platelet count greater than 80 × 10^9^/l and biochemical parameters were within normal range. Nadir blood counts were not routinely measured. All patients received growth factor support with pegylated granulocyte colony stimulating factor given within 72 h of last chemotherapy. Toxicity was assessed using the Common Terminology Criteria for Adverse Events (CTCAE) Version 3.0. In the event of lack of recovery from toxicity, chemotherapy was delayed or modified at the discretion of the treating physician. Radiological response assessment was carried out with restaging CT and MRI scans after 4 and 8 cycles of treatment. Fourteen cycles of chemotherapy were planned subject to tolerability and response, with treatment interrupted after 6 cycles or 12 weeks for surgery if applicable. When radiotherapy was the primary modality for local control, this was given concurrently with chemotherapy beginning after cycle 6 and with the omission of anthracyclines from relevant cycles.

## Results

### Patient characteristics

Between September 2008 and October 2011, 16 patients received 193 cycles of interval-compressed VDC/IE (Table 1). Ten patients had EFT, two had UHGRCS, and four patients had DSRCT. Fourteen (88%) had metastatic disease and 11 patients had more than one site of metastasis. The remaining three had metastatic disease confined to the lung. Seventy per cent of patients were older than 18 years (median 22 years).

**Table 1:** Patient demographics

	**No. of patients (n = 16)**	**%**
**Age in years**		
Median (range)	22 (14–37)	100%
14-17	4	25%
18-25	7	44%
25-37	5	31%
**Sex**		
Male: Female	12: 4	
**Diagnosis**		
EFT	10	62.5%
DSRCT	4	25%
UHGRCS	2	12.5%
**Primary Site**		
**Ewing’s Sarcoma**	10	
Pelvis	5	50%
Kidney	1	10%
Lower extremity	2	20%
Rib	1	10%
Unknown	1	10%
**UHGRCS**	2	
Pelvis	1	50%
Mediastinum	1	50%
**Extent of disease**		
Localised	2	12%
Metastatic	14	88%
Bone only	5	36%
Lung and bone	3	21%
Lung, bone, BM	2	14%
Bone and BM	1	7%
Lung only	3	21%

UHGRCS,Undifferentiated high grade round cell sarcoma; DSRCT, Desmoplastic small round cell tumour; BM, bone marrow.

#### Treatment

Eight patients completed 14 cycles of VDC/IE (Table 2). Six patients discontinued VDC/IE early, 5 due to toxicity and one because of progression. These six received a median of 11 cycles (range 4–12). Patient 3 had early surgery after 4 cycles having experienced severe haematological and gastrointestinal toxicity. Chemotherapy was not continued post operatively and the patient remains disease free at 2 years. Patient 11 discontinued after cycle 11 due to prolonged thrombocytopenia following pelvic irradiation. Patient 2 completed 12 cycles and then developed a deep infection around a pelvic spacer inserted for radiotherapy. Patients 14 and 15 also completed 11 and 12 cycles respectively experiencing severe cumulative fatigue and haematological toxicity. Patient 5 had progressive disease after 11 cycles. Two patients with EFT were switched to receive VDC/IE having previously commenced alternative chemotherapy regimens and did not complete all 14 cycles. Patient 6 had 3 cycles of VIDE followed by 8 cycles of VDC/IE and patient 16 had a cycle of VAC (vincristine, actinomycin-D, cyclophosphamide), then VID (vincristine, ifosfamide, doxorubicin) followed by 12 cycles of VDC/IE.

**Table 2:** Demonstrates the treatment received by each patient including the local treatment and outcome to date

**Patient No.**	**Age at diagnosis**	**Gender**	**Diagnosis**	**Primary site**	**Site of metastases**	**Prior chemo**	**No of VDC/IE cycles**	**Cycle treatment Interval (days)**	**Dose reduction**	**Local treatment**	**Outcome**
1	14	F	EFT	Kidney	Bone, peritoneum	No	14	19.9	Yes	Surgery, R1 resection, poor response to chemo on histology	Relapsed 3 months after completing VDC/IE – receiving palliative chemotherapy
2	22	M	EFT	Ilium	Lung, bone, BM	No	12	17.1	Yes	RT - 50Gy in 30# 2 phases	Relapsed 10 months post treatment and died 23 months post treatment
4	29	M	EFT	Femur	Bone, BM	No	14	14.9	Yes	Surgery, R0 resection with good response to chemo on histology	Relapsed 7 months post treatment and died 11 months after treatment
5	15	F	EFT	Unknown	Lung, Bone, BM	No	11	17.5	Yes	None	Progressed at cycle 11 and died 3 months later
6	17	M	EFT	Ilium	Bone	Yes	8	15.4	No	RT 55Gy in 31#	Relapsed 4 month post treatment. Died 15 months after completing treatment.
7	29	M	EFT	ilium	Lung, Bone	No	14	16.5	Yes	RT 45Gy in 25#	Progressed at the end of treatment and died 3 months later
11	21	M	EFT	Sacrum	bone	No	11	17.4	No	RT 50.4Gy in 28# in 2 phases to sacrum and 55Gy in 30# to chest	Relapsed 4 months post treatment and died 6 months post treatment
12	19	M	EFT	Pelvis	Bone	No	14	16.5	No	RT 55Gy in 30#	Disease free at 4 months post treatment
15	19	M	EFT	Metatarsal	Lung, Bone	No	12	17.9	No	Surgery, close margins, poor response to chemo on histology	Relapsed in lung 11 months treatment and then lost to follow up in 8 months after relapse as living abroad.
16	17	M	EFT	First rib	Bone	Yes	12	18.5	Yes	RT 55.8Gy in 31#	Died of disease 4 months after completing treatment.
3	37	F	UHGRCS	Ilium	none	No	4	16.2	No	Surgery, R0 resection. Poor response to chemo on histology	Disease free 25 months post treatment
14	27	F	UHGRCS	mediastinum	None	No	11	19.9	Yes	Surgery, R0 resection with excellent response to chemo on histology	Disease free 1 month post treatment
8	24	M	DSRCT	n/a	Peritoneal cavity, lungs	No	14	15.9	No	None	Progressed 5 months post treatment, while on maintenance VAC chemotherapy and is having further palliative chemo
9	31	M	DSCRT	n/a	Peritoneal cavity Lung, bone	No	14	15	No	None	Progressed 4 months post treatment and is currently having further palliative chemo
10	19	M	DSCRT	n/a	Peritoneal cavity, liver	No	14	14.9	No	None	No progression 6 months post treatment on maintenance VAC
13	22	M	DSCRT	n/a	Peritoneal cavity	No	14	14.9	No	None	No evidence of progression one month post treatment

M, Male; F,Female; EFT, Ewing’s family of tumors; UHGRCS,Undifferentiated high grade round cell sarcoma; DSRCT, Desmoplastic small round cell tumour; RT, radiotherapy; R1, microscopically positive margins; R0, complete excision.

#### Treatment interval

**Figure 1:**
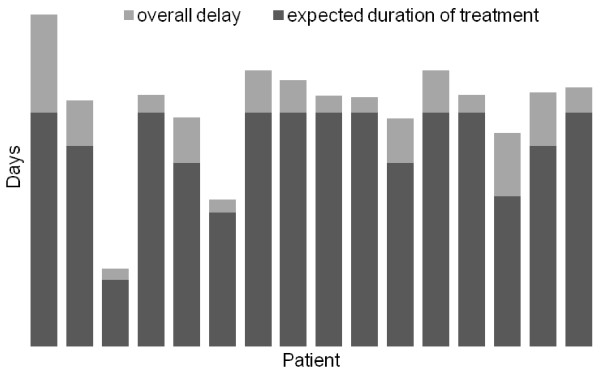
**Duration of treatment.** The expected duration of treatment for each patient in days, determined by the number of cycles received (dark grey), and the additional days required to complete treatment (light grey).

**Figure 2:**
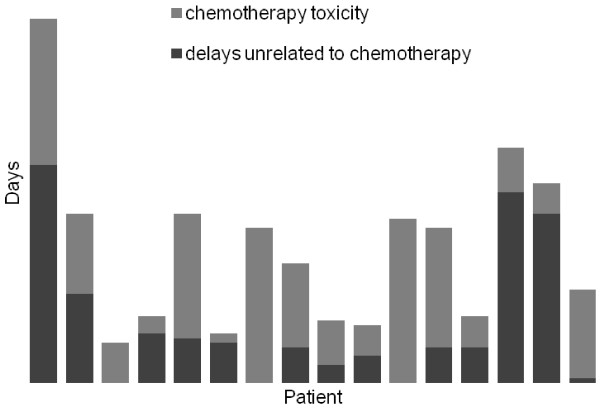
**Reasons for Delay.** Delay in days is divided between chemotherapy related toxicity (light grey) and other factors such as interruptions for surgery (dark grey).

The mean treatment interval for each patient was calculated from the entire treatment duration in days divided by the number of cycles that were completed in this time. The mean interval between cycles was 16.7 days (range 14 to 57 days). All 16 patients took longer than expected to complete the treatment course. Figure 1 demonstrates the total number of days delay over the entire treatment schedule for each patient. The number of days that treatment was delayed due to chemotherapy related toxicity and other causes are demonstrated for each patient in Figure 2. The main chemotherapy related cause for delay was neutropenia, febrile neutropenia and thrombocytopenia. Of the non-chemotherapy related causes, most delays were due to surgery. Chemotherapy was continued during and after radiotherapy in all but one patient who developed prolonged thrombocytopenia following pelvic radiotherapy.

#### Dose modifications

Nine patients (56%), including 5 of the 8 who completed all 14 cycles, had no dose reduction, whereas 7 patients required varying dose reductions, as follows. Patient 1 required a 50% dose reduction of vincristine alone at cycle 3 due to grade 3 neuropathy but later recovered and received full dose vincristine for all future cycles. However she required a 20% dose reduction of ifosfamide and etoposide at cycle 8 due to grade 3 thrombocytopenia, anaemia and mucositis. The dose reduction continued for cycle 10 and a further 20% reduction was made at cycle 12 for on-going grade 3 bone marrow toxicity but the patient completed 14 cycles. Patient 2 had a 20% dose reduction of doxorubicin and cyclophosphamide at cycles 7, 9 and 11. At cycle 10 a 20% reduction of Ifosfamide and etoposide was added which continued for cycle 12. All reductions were due to episodes of febrile neutropenia and fatigue. Treatment was terminated at cycle 12 due to a prolonged infection surrounding his pelvic spacer. Patient 4 had a 20% dose reduction of doxorubicin alone at cycles 9 and 11 due to febrile neutropenia. Doxorubicin was omitted for cycle 13 as the patient was receiving concurrent radiotherapy. Patient 5 had the first cycle of VDC with a 20% reduction of all three drugs due to poor performance status. Subsequent cycles were given at full dose till cycle 7 when vincristine was dose reduced by 50% due to grade 3 peripheral neuropathy. This reduction continued for cycle 9 and 11 when treatment was discontinued due to progressive disease. Patient 7 had a 20% dose reduction of doxorubicin at cycle 13 alone due to a grade 3 thrombocytopenia and neutropenia. He completed 14 cycles in total. Patient 14 experienced grade 3 peripheral neuropathy at cycle 3 which led to a 50% dose reduction of vincristine and subsequent omission from a further 4 cycles of VDC. By the end of treatment, the neuropathy had improved to grade 2. No other dose modifications were made and she requested discontinuation of treatment at cycle 11 due to cumulative fatigue and haematological toxicity. Finally, patient 16 had a 20% dose reduction from cycle 5 in all drugs except vincristine because of grade 3 thrombocytopenia and mucositis.

#### Adverse events

There were no toxicity related deaths. The rates of grade 3 and 4 toxicities per cycle are illustrated in Table 3. Grade 3 febrile neutropenia was observed in 26 cycles (13.5%) and there were 6 episodes of non-neutropenia related infection requiring admission to hospital. Grade 3/4 anaemia was recorded in 31 cycles (16%) and thrombocytopenia in 25 cycles (13%) on routine pre-chemotherapy blood counts performed on day 14. Grade 3 or 4 mucositis was observed in only 12 cycles (6.2%). There were no grade 3 and 4 cardiac or renal toxicities and no episodes of grade 3 and 4 encephalopathy.

**Table 3:** Number of cycles complicated by grade 3 or 4 toxicity out of the total of 193 delivered cycles of VDC/IE

**Grade 3 & 4 Toxicity rates/193 cycles**	**Grade 3**	**Grade 4**	**Total Grade 3 and 4 (%)**
Febrile Neutropenia	26	0	26 (13.5)
Non-febrile neutropenia	3	11	14 (7.3)
Anaemia	29	2	31 (16.1)
Thrombocytopenia	19	6	25 (13.0)
Mucositis	12	0	12 (6.2)
Non-neutropenic infection requiring hospital admission	6	0	6 (3.1)
Neuropathy	3	0	3 (1.6)
Fatigue	3	0	3 (1.6)
Diarrhoea	2	0	2 (1.0)
Nausea and vomiting	2	0	2 (1.0)

#### Outcome

All 10 patients with EFT had a radiological response to therapy (Table 2). Nine patients subsequently had local treatment to the primary site. Three patients had surgery, with a good response to chemotherapy (>90% necrosis) evident in one. The remaining six patients had radiotherapy to the primary site. The tenth patient with EFT had widely metastatic disease with no identifiable primary tumour. Nine patients have relapsed or progressed between 1 and 11 months after completing treatment and seven have died.

The four patients with DSCRT all completed 14 cycles of treatment. All had clinical benefit from chemotherapy.

Two patients with high grade undifferentiated round cell tumours were treated. The first had stable disease after 4 cycles of VDC/IE and underwent surgery with a poor histological response. This patient remains progression free after 25 months. The second patient underwent surgery after 8 cycles, with a complete histological response and is free from progression 3 months after completion of chemotherapy.

## Discussion

In response to emerging data from the US indicating that interval compressed VDC/IE was a well-tolerated, effective regimen for EFT, we selected patients who were ineligible for randomisation in Euro EWING 99 to receive this treatment in preference to the institutional standard, VIDE [16,21]. We wished to gain preliminary experience of the feasibility of this regimen and extend its use to a population with metastatic disease and older patients, groups not represented in the randomised study from COG. The results indicate that this regimen can be safely delivered and interval compression achieved in this patient group. Although this is a small study, these data also lend support to the view that this is a tolerable regimen, even in an older patient group with considerable disease burden. Finally, we observed clinical benefit of dose compressed VDC/IE in DSRCT.

Toxicities associated with VIDE are well recorded both from the initial single institution study [21] and from a large analysis of the first 851 patients included in EURO-E.W.I.N.G. 99 [16]. A total of 4,746 courses of VIDE in 851 patients were analysed with respect to toxicity. The rate of febrile neutropenia was 60.8% with, and 65.8% without, GCSF support.

Toxicity data from the AEWSOO31 study is currently available only in abstract but reports a rate of grade 3/4 febrile neutropenia of 6.9% in the two weekly arm [18]. While emphasising the very small number of patients reported here, this figure is lower than that reported here (13.5%). However, the several differences in patient demographics may account for this. Firstly the AEWS0031 study was in a paediatric population with only 75 of the 587 (12.8%) patients over the age of 18, whereas in this study 12 patients were over the age of 18 (75%). Secondly, the AEWS0031 study was carried out in patients who did not have metastatic disease. Again, in our cohort, the majority of patients had metastatic disease and a large overall tumour burden, 11 (69%) patients having bone, bone marrow and lung metastases, and 7 (44%) with primary pelvic disease.

The determinants of chemotherapy tolerance in EFT require further clarification. Comparisons of the influence of factors such as age are limited by the absence of planned prospective analyses and the reporting of cohorts containing varying proportions of children, adolescents and adults. Hence studies that report the adverse influence of younger age may contain few adults [22]; others focus exclusively on much older adults [23]; while still others fall between these extremes [19,24]. In the largest study, from EURO-E.W.I.N.G. 99, chemotherapy toxicity was not clearly greater in older patients but dose modifications were more frequent [16]. Most clinicians will remain cautious when treating older patients or those with heavy tumour burden with the current intensive regimens discussed here.

The treatment interval for the two weekly regimen was successfully maintained in this patient cohort with a mean treatment interval of 16.7 days. This resulted in a shorter overall treatment time when compared to a 3-weekly regimen. For the 8 patients who completed all 14 cycles of treatment the average overall treatment time was 253 days, which is markedly shorter than the 294 days it takes to complete standard treatment with VIDE/VAI. In the EURO-E.W.I.N.G 99 clinical trial, dose modifications were recorded in 1020 of the 4746 courses (21%) of all cycles. In this study, only 26 cycles were given at a reduced dose (14%), therefore we can postulate that dose intensity was not compromised in order to maintain a shorter dose interval. Three patients terminated treatment early due to chemotherapy-related toxicity; two at cycle 11 and one at cycle 12.

The main limitation of this study is that it is a small non-randomised cohort of patients with heterogeneity of presenting clinical features. The poor prognostic factors present in several patients and the need to gain familiarity with the regimen will have had an effect on the application of clinical thresholds for decisions on dose reductions and cessation of treatment. The information on clinical outcome is provoking but should be considered only with caution. Despite this, the data do have value in supporting the premise that this may be a less toxic regimen than VIDE, which is deliverable in patients outside of the limited eligibility criteria for AEWS0031.

In conclusion, this study demonstrates that interval compression of chemotherapy is feasible in an older and higher risk cohort of patients with EFT and other small round cell sarcomas. This schedule appears well tolerated compared with the standard European treatment of VIDE followed by VAC or VAI. We also included patients who would traditionally have a poor long term survival even with intensive chemotherapy. A shorter, less toxic regimen is an attractive option for these patients in whom life expectancy is likely to be limited by their disease. Further data regarding efficacy and long term toxicity will be available in the future as this regimen will now be compared with the VIDE/VAI schedule in a multi-centre randomised controlled trial.

## Competing interest

The authors declare that they have no competing interest.

## Authors’ contributions

JW conceived the study; analysed and interpreted data; drafted the manuscript. AK, AS and CR collected, analysed and interpreted the data and contributed to drafting the manuscript. BS, MM, SS contributed to the study design, collection and interpretation of data and contributed to the manuscript. All authors read and approved the final manuscript.
